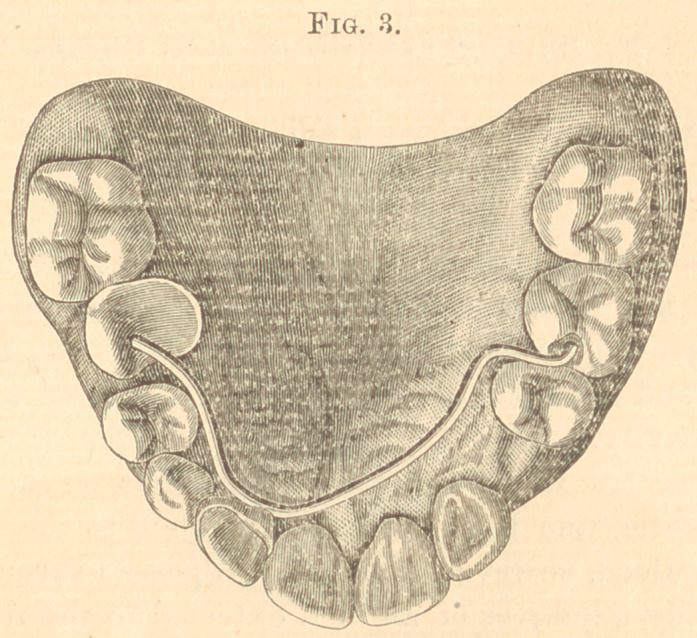# Some Methods of Making and Retaining Removable Appliances for Correcting Irregularities of the Teeth

**Published:** 1890-04

**Authors:** V. H. Jackson

**Affiliations:** New York


### SOME METHODS OF MAKING AND RETAINING REMOVABLE APPLIANCES FOR CORRECTING IRREGULARITIES OF THE TEETH.[1]

BY V. H. JACKSON, D.D.S., NEW YORK.

Gentlemen of the Union Meeting,—May I have your attention to consider some methods of making and retaining appliances for moving teeth and how to make additions to apparatus already in use, which have been used satisfactorily in my practice.

As bands of gold and gold and platinum are being used generally for attaching and retaining appliances to the teeth, and also for retaining teeth in position after regulating, a few remarks may be appropriate first, as to the best method of making and retaining them.

The metal should be as thin as can be used, and still have sufficient rigidity so that when driven it will conform readily to the tooth.

Gold meets the requirements better than gold and platinum in most cases, as the latter is not sufficiently yielding, and is more liable to discolor when used in contact with piano wire. Better results are obtained by forming the band on the natural tooth than on a model, and much depends on its adaptation.

If there is to be much strain on the band it should be rather broad, and burnished to the tooth with the ends lapping on the lingual side in most cases, at an angle best suited to make a good adaptation. For the incisors or cuspids, a broad piece of gold may have a V-shaped piece cut out of either end, and then burnished to

---

[1] Read before the Union meeting held at Springfield, Mass., October 24, 1889.

the tooth with the ends drawn together on the lingual surface and soldered, thus adapting it perfectly to the tooth.

A very strong band can be made by fitting two narrow bands to the tooth, having them close together on the lingual and separated on the labial side, and make an impression with mouldine of the front of the tooth and bands, then remove the bands and place them in the impression and solder the parts to be united.

The bands can be used in this way, or a thin piece of gold can be burnished to the labial side of the band and soldered.

To assist in retaining, the inside of the band should be roughened by raising ridges with a sharp instrument, and the tooth polished with a fine grade of pumice-stone so the cement will adhere more readily to it. The cement is used rather thin, and kept dry by rubber dam or spunk.

Where there is a great downward pressure on the band, as when superior incisors and cuspids are being forced forward, it can be sustained in many cases by passing a small platinum wire around the neck of the tooth one or more times, then passing the ends either way below the band, and then twisting together before the cement hardens. If well adjusted this will in most cases resist the most severe strain on the band.

There is also a method of putting a screw through the band and adjusting with zinc phosphate, at the same time tightening the screw.

If there is to be a projection from the band on the distal or mesial surface, for the purpose of retaining a tooth once rotated, or to hold an appliance from slipping from the lingual surface of the incisors, it can be most easily made by bending the ends at a right angle with the bands and then soldering.

The projecting end can be ground or filed to any desired shape, or a lug, or tube, may be soldered to the band for similar purposes.

Bands can be removed usually without cutting, by forcing a thin straight burnisher between the band and tooth.

It frequently occurs, when regulating with rubber plates, that an extra spring is needed to complete the regulating. The writer has often attached a piece of piano wire for that purpose, by passing it through a hole in the plate a quarter or half an inch, with the end flattened and formed to fit the surface, and fastened by drawing a binding wire through holes in the plate either side of the flattened wire, and twisting the ends together.

Doubtless a number of the gentlemen present heard a paper read before the American Dental Association in August last, in which I described a method of uniting piano wire to form independent appliances for the purpose of moving teeth.

Two or more pieces of piano wire are joined in any desired position, and held temporarily with cord, then bound with fine binding wire of copper or iron, wound close together usually, and soldered with soft solder or tin, by holding the parts over a spirit lamp and applying small pieces of solder, the surface having been covered with muriate of zinc; the zinc solution should be very much diluted.

The method of attaching springs to a rubber plate is accomplished in a similar manner by soldering a piece of metal to the end of a piano wire, to be used as a spring for moving teeth, and for retaining the plate in position in the mouth, etc. (See Fig. 1.)

Fig. 1.

Reduced one-half.

The spring or piano wire is flattened on the end, without drawing the temper, and a thin piece of coin silver, German silver or tinned copper, about one-fourth by half an inch in size, is made in form something like the bowl of a spoon. The flattened end of the wire is then fastened into the depression of the metal by drawing binding wire through holes made with the plate punch, either side of the piano wire, and twisted as close as practicable, with the ends left long, and coiled up in the depression to assist the solder in flowing.

It is then heated over a spirit lamp and soldered by applying pieces of solder or tin, as before described, until the bowl part is filled, when the surface of the solder may be filed, or flattened by turning it down on an anvil and cover with a thick piece of lead and hammered until it is level; the latter will be found the quicker method.

The edge of the silver is trimmed to the desired form, and holes punched with the plate punch in the corners for the rivets.

Gold can be used in place of other metals, if soft solder is used, but, owing to the great affinity of tin and gold, tin is not practicable.

A piece of watch- or clock-spring can be used to advantage in some cases in place of piano wire.

The common brass pin with the temper drawn is a convenient rivet to fasten springs to rubber plates.

The whole appliance can be immersed in molten tin, if it is desired, soldering and plating it at once, or the wire can be tinned before soldering.

In this manner bands or clasps of silver or most any metal can be attached to the end of spring wire to hold it in place on any tooth it is desired to move.

Fig. 2.

A method of keeping the end of the spring wire from pressing on the gum, or slipping off of the teeth when regulating molars or bicuspids, is accomplished by twisting a copper wire around the piano wire one or more times, and soldering with soft solder. Apply by allowing one end to project into the space between the teeth, and the other end to rest on the grinding surface.

For the same purpose a bow of copper wire may extend up over any prominence on the surface of a tooth with the ends united to the piano wire opposite the space between the teeth, as before, or a very thin piece of tinned copper may be burnished to the model of the tooth and soldered to the spring wire in the manner described for soldering metal to piano wire.

The practicability of spring wire being used independently of plates or fixed appliances I have demonstrated in many cases, as from my experience I have found but few irregularities of the teeth that cannot be corrected by the use of the band and spring, which, as a rule, is more easily made and adjusted than other appliances.

Fig. 3.

A simple method adopted to force a superior incisor into proper position that stood inside the normal line of the arch was to cement a band to the tooth, with a U-shaped piece of metal attached to the lingual side. A piano wire of about No. 20 gauge was formed to follow the curve of the arch back of the incisors with the ends in form of a letter S and allowed to project into anterior proximal cavities of the temporary molars. (See Figs. 2 and 3.)

The pressure was increased by removing the wire and straightening the ends. The regulation of the tooth was accomplished in thirty-four days, with perfect comfort to the patient, a very desirable feature, especially in the case of children.

## Figures and Tables

**Fig. 1. f1:**
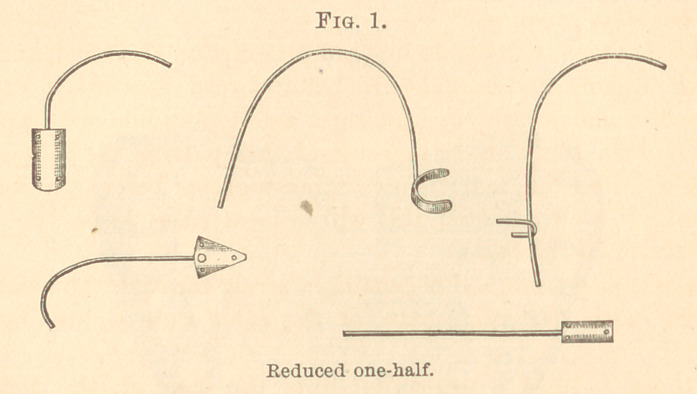


**Fig. 2. f2:**
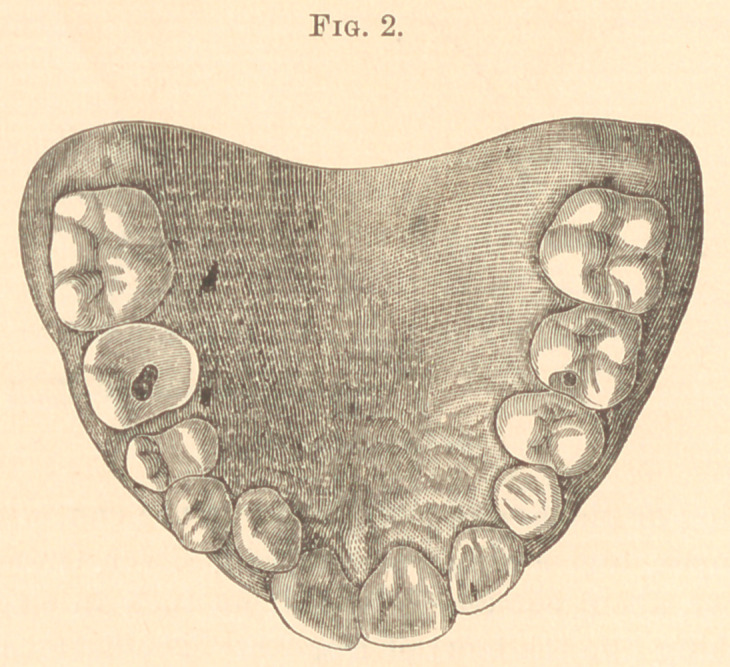


**Fig. 3. f3:**